# An EMT-based gene signature enhances the clinical understanding and prognostic prediction of patients with ovarian cancers

**DOI:** 10.1186/s13048-023-01132-2

**Published:** 2023-03-13

**Authors:** Qi-jia Li, Zi-liang Wu, Juan Wang, Jing Jiang, Bing Lin

**Affiliations:** 1 Hospital of Chengdu University of Traditional Chinese Medicine, No.39 Shi-er-qiao Road, Chengdu, 610072 Sichuan Province China; 2 Department of Public Health, School of Clinical Medicine, Chengdu University of Traditional Chinese Medicine, Chengdu, 610072 China

**Keywords:** Ovarian cancer, EMT, Prognosis, Immune infiltration, Chemotherapy

## Abstract

**Background:**

Ovarian cancer (OC) is one of the most common gynecological cancers with malignant metastasis and poor prognosis. Current evidence substantiates that epithelial-mesenchymal transition (EMT) is a critical mechanism that drives OC progression. In this study, we aspire to identify pivotal EMT-related genes (EMTG) in OC development, and establish an EMT gene-based model for prognosis prediction.

**Methods:**

We constructed the risk score model by screening EMT genes via univariate/LASSO/step multivariate Cox regressions in the OC cohort from TCGA database. The efficacy of the EMTG model was tested in external GEO cohort, and quantified by the nomogram. Moreover, the immune infiltration and chemotherapy sensitivity were analyzed in different risk score groups.

**Results:**

We established a 11-EMTGs risk score model to predict the prognosis of OC patients. Based on the model, OC patients were split into high- and low- risk score groups, and the high-risk score group had an inevitably poor survival. The predictive power of the model was verified by external OC cohort. The nomogram showed that the model was an independent factor for prognosis prediction. Moreover, immune infiltration analysis revealed the immunosuppressive microenvironment in the high-risk score group. Finally, the EMTG model can be used to predict the sensitivity to chemotherapy drugs.

**Conclusions:**

This study demonstrated that EMTG model was a powerful tool for prognostic prediction of OC patients. Our work not only provide a novel insight into the etiology of OC tumorigenesis, but also can be used in the clinical decisions on OC treatment.

**Supplementary Information:**

The online version contains supplementary material available at 10.1186/s13048-023-01132-2.

## Background

Ovarian cancer (OC) is one of the most fatal and lethal gynecologic cancers for women [1, 2]. There are more than 295,000 newly diagnosed OC patients and 184,000 deaths per year [3]. Histologically, OC can be split into epithelial, germ cell, and sex-cord-stromal subtypes, among which the epithelial subtype occupies the vast majority (> 90%) [4, 5]. Epithelial OC originates from the ovarian surface epithelium and fallopian tubes [6], with the 5-year survival rate < 50% after diagnosis [7]. A wide range of genetic and environmental factors can increase the risk of OC, including aging, genomic mutation [8], ovulation [9], postmenopausal hormonal therapy use [10], infertility and nulliparity [11]. Despite advanced medical and surgical treatments, including debulking surgery, neoadjuvant therapy, and chemotherapy [12], the long-term survival for OC patients remains not satisfied, mainly due to the failure to detect the tumor at early-stages. In fact, over 70% OC are diagnosed at Stage III or IV [13], with inevitably poor prognosis. Accordingly, there is increasing pressure on developing prognostic models for the survival prediction of OC.

Over decades, preclinical studies have revealed the pathological origins, cellular heterogeneities, and molecular features of OC, with implications in clinical benefit [14]. The invasive characteristic of OC is drived by multiple cellular events, including epithelial-mesenchymal transition (EMT) [15]. EMT is defined as a cellular event characterized by epithelial cells acquiring mesenchymal phenotypes in parallel with loss of epithelial features [16, 17]. Once EMT programs are activated, tumor epithelial cells abolish their polarity and cell-cell adhesion, and gain invasive abilities [18]. Emerging evidence has suggested that EMT not only promotes tumor metastasis, but also increases tumor stemness, cytokine release, cancer-associated angiogenesis, immune escapes, and chemoresistance [18]. Therefore, EMT is widely accepted as a hallmark of cancer [19]. Current understandings have described multiple factors regulating EMT in OC, including LEFTY1 [20], TRAP1 [21], MDM2 [22], and Rac1 [23]. All these experimental data provide the possibility to build EMT gene (EMTG) models for prognosis prediction of OC.

**Fig. 1:**
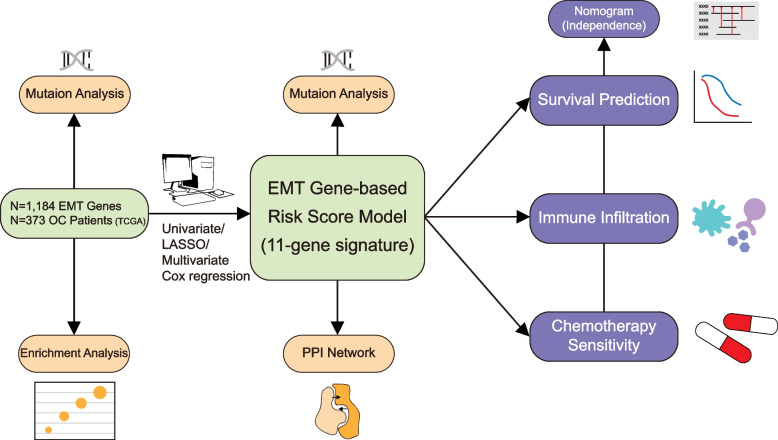
Graphic workflow of this study. A flow chart of this study showing the procedure of identification and exploration of 11-EMT gene-based risk score model in OC

In this study, we performed comprehensive analysis to establish an EMTG-based risk score model to predict the survival status of OC patients, using the EMTG expression files from public database. Moreover, we applied our EMTG model to evaluate the immune infiltration and chemotherapy sensitivities (Fig. 1). Our work demonstrates the prognostic power of EMTG in OC, and affords a potential tool for improving OS management.

## Methods

### Dataset and clinical information acquisition

The EMT gene set was obtained from dbEMT database with a total of 1184 EMT-related genes [24, 25]. The transcriptomic files and clinical information were collected from the Cancer Genome Atlas (TCGA) portal (https://portal.gdc.cancer.gov/) and GEO database (https://www.ncbi.nlm.nih.gov/geo/). The TCGA-OC cohort included 373 patients was assigned as the training set, while the GSE26193 testing set contained 107 OC patients.

### Functional enrichment and genomic mutation analysis

The Gene Ontology (GO) and Kyoto Encyclopedia of Genes and Genomes (KEGG) pathway enrichment analyses were conducted to demonstrate the EMT gene related biological functions using the “clusterprofiler” package of R software. For mutation analysis of the whole genome or EMT genes, somatic mutation data of OC were downloaded from the TCGA and analyzed by the “maftools” R package. For protein-protein interaction (PPI) analysis, the GeneMANIA database was applied to construct PPI network [26].

### Identification and verification of the risk score model

To establish the EMT gene-based model, 1184 EMT-related genes were screened by the univariate Cox regression analysis using “survival” and “survminer” R packages (*p* < 0.05) [27], and 96 genes were retained. The 96 genes were crossed with the differentially expressed genes (DEGs, *n* = 5520) between OC patients and controls, to get a set of 23 genes. Next, the LASSO Cox analysis (by the “glmnet” R package [28]) was conducted to get 16 genes for step wise multivariate Cox regression analysis (by “survival” and “survminer” R packages [27]). Finally, 11 genes were obtained to construct the EMT gene risk score model, and the formula to calculate the probability of inferior survival for each sample was, Risk score = expression of gene 1 × β1 + expression of gene 2 × β2 + ⋯ + expression of gene n × βn, (β indicating the coefficients).

Then, the TCGA-OC cohort were divided into low- and high-risk groups according to the median risk score. The Kaplan–Meier (K-M) survival analysis was adopted to compare the survival difference between the two groups. The time-dependent receiver operating characteristic (ROC) analysis (by “timeROC” R package) was used to assess the discrimination of the risk score model. Moreover, the univariate and multivariate Cox analyses were performed to evaluate the independence of risk scores and the prognosis of OC patients. Finally, to verify the efficacy of the risk score model, the above process was applied in additional testing cohort from GSE26193.

### Construction of the risk score-based nomogram

An EMT gene-based nomogram was conducted to predict the survival rate of individual OC patient. Clinical factors including the age, disease grade, histological stage and risk score were incorporated using the “rms” package of R software.

### Evaluation of the immune infiltrations

The CIBERSORT algorithm was used to evaluate infiltrations of 22 immune cells for each OC patient in TCGA-OC cohort, using the “CIBERSORT” R package [29]. All the TCGA-OC patients were divided into the low- and high-risk score groups to compare the difference in immune cell compositions. The *p* value of each sample deconvolution was determined by CIBERSORT, and samples with *p* < 0.05 was screened out for box-plot graphing.

### Correlation analysis of the risk score with drug sensitivity

To study the relationship between the risk score and drug sensitivity, the CellMiner database was applied (v2.7, https://discover.nci.nih.gov/cellminer/home.do) [30]. The transcriptomic data of 60 cancer cell lines and the IC50 of over 20, 000 drugs (approved by FDA or under clinical trials) were interrogated to determine the correlative significance with the |cor| > 0.3 and *p* < 0.01, and visualized by scatter plot using the “ggplot2” R package, which is performed by a well-established pipeline in [31–33].

### Statistical analysis

All statistical analyses were performed by the R software (version 4.0.3). The statistical significance was established with the *p* value < 0.05 without additional statement.

## Results

### Functional enrichment and genomic mutation of EMT genes related to OC

**Fig. 2:**
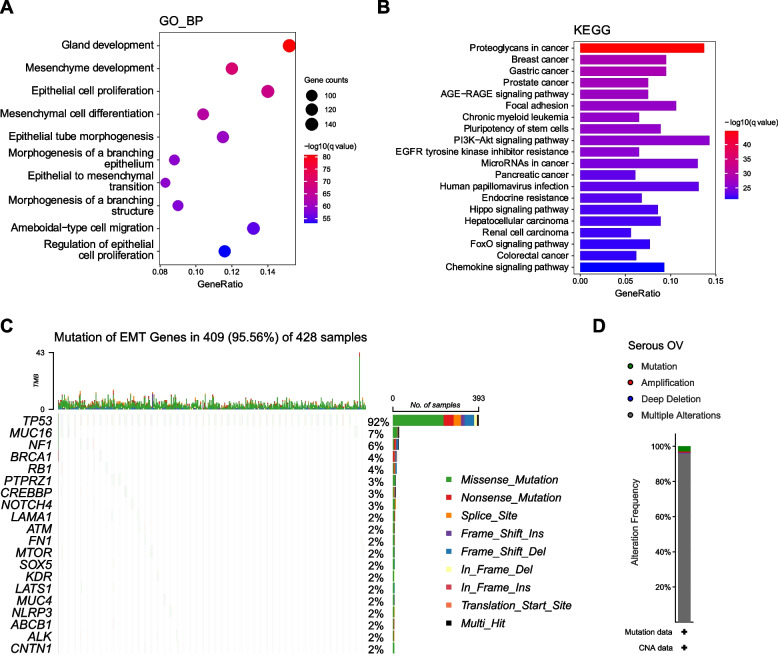
Functional enrichment and genomic mutation of EMT genes related to OC. **A-B** GO (**A**) and KEGG (**B**) analysis showing the functional enrichment of EMT genes. **C** The waterfall plots showing the top 20 mutated EMT genes in the OC cohort of TCGA. Each row represents a mutated gene, and each column represents a patient. **D** The alteration frequency of EMT genes in OC patients from cBioPortal database

To deeply explore the EMT gene (EMTG) alterations in OC, we investigated their biological functions and genomic features. First, we applied GO and KEGG analysis to detect the functional enrichment of 1184 EMTG. Results of the GO analysis showed that the top terms were enriched in tissue development and cell proliferation, including mesenchyme development, epithelial cell proliferation and mesenchymal cell differentiation (Fig. 2A). KEGG analysis further demonstrated that the enriched pathways were consisted of multiple cancer items, focal adhesion, oncogenic PI3K/AKT, Hippo and FoxO signalings (Fig. 2B), supporting the notion that EMT is correlated with multiple human cancers and interconnected with oncogenic pathways.

Next, we examined the mutation frequency of 1184 EMTG in TCGA-OC cohort, to address whether genomic mutation is a leading cause of the EMT in OC. It’s found that EMTG mutation occurred in 95.56% OC patients, and the top 20 mutation frequency ranging from 2 to 92% (Fig. 2C). The mutation frequency of *TP53* reached 92%, which may be enriched in the Type II of epithelial ovarian cancers [34, 35]. However, the mutation frequency of other genes was lower than 10%, including *BRCA1* (4%), *ATM* (2%), *MTOR* (2%), and *LATS1* (2%) (Fig. 2C and Supplementary Fig. S1). Similarly, results of the cBioPortal database showed that the major mutation style of the EMTG was Multiple Alterations, not Amplification or Deep Deletion (Fig. 2D and Supplementary Fig. S1). Thus, most EMTG (except *TP53*) tend to preserve genomic stabilities. All these results suggest that altered EMTG expression, but not genomic mutation, may be the major cause of dysregulation of EMTG in OC.

### Identification and validation of the EMTG risk score model in OC

**Fig. 3:**
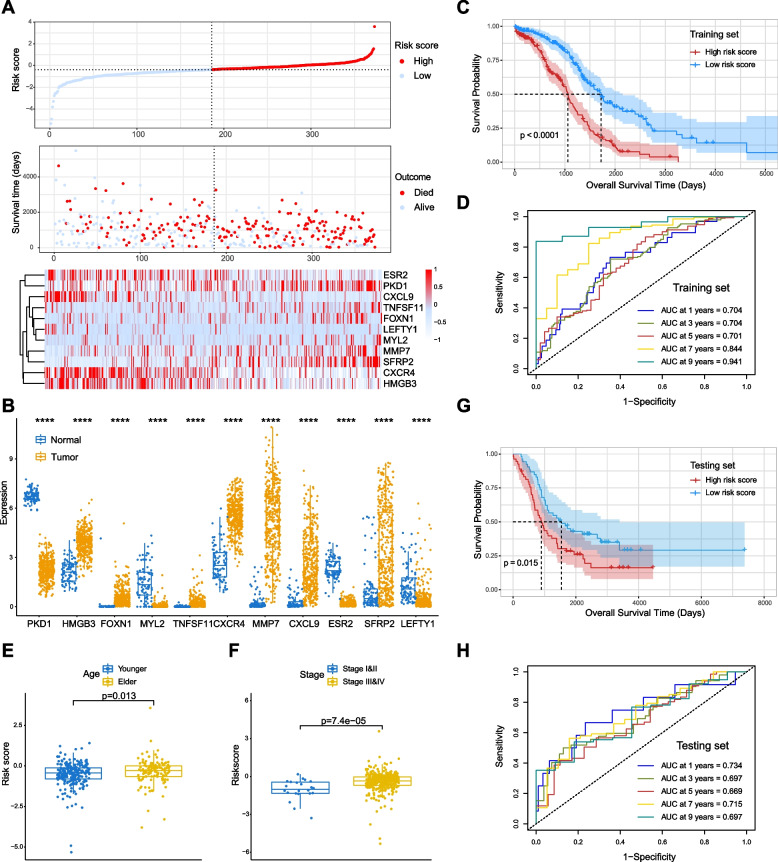
Identification and validation of the EMTG risk score model in OC. **A** The heatmap showing the distribution of 11 EMTG genes and survival status for each patient of OC in TCGA (training set). **B** The box plots showing the 11 EMTG expression levels in tumors and controls in TCGA. **C** Kaplan–Meier analysis showing the distinct survival tendency in low- and high-risk score groups in TCGA (training set). **D** ROC curves showing the predictive efficiency of EMTG model for the survival rate in TCGA (training set). **E-F** Diagrams showing the correlations of risk score with patient age (**E**) and histological stages (**F**) in TCGA cohort. **G** Kaplan–Meier analysis showing the distinct survival tendency in low- and high-risk score groups in GSE26193 (testing set). **H** ROC curves showing the predictive efficiency of EMTG model for the survival rate in GSE26193 set

Based on above analysis, we constructed the risk score model, by importing EMTGs into a multiple step screening pipeline including the univariate Cox regression, LASSO Cox regression and multivariate Cox analysis with stepwise regression in TCGA-OC cohort (Fig. 1 and Supplementary Fig. S2). Afterwards, the 11-gene EMTG signature were obtained comprised of *ESR2*, *PKD1*, *CXCL9*, *TNFSF11*, *FOXN1*, *LEFTY1*, *MYL2*, *MMP7*, *SFRP2*, *CXCR4*, and *HMGB3* (Fig. 3A). All the 11 EMTG were differentially expressed between tumor tissues and normal controls. Evidently, expressions of *HMGB3*, *FOXN1*, *TNFSF11*, *CXCR4*, *MMP7*, *CXCL9* and *SFRP2* were higher in tumors, while *PKD1*, *MYL2*, *ESR2* and *LEFTY1* were higher in the normal controls (Fig. 3B).

Next, the Kaplan-Meier (K-M) survival analysis revealed that the overall survival (OS) of the low-risk score group was much better than the high-risk group (Fig. 3C). The ROC analysis confirmed the predictive ability of the EMTG signature, showing the AUC values of 0.704 (1-year), 0.704 (3-year), 0.701 (5-year), 0.844 (7-year), and 0.941 (9-year) respectively (Fig. 3D). Moreover, we split the OS patients into distinct subgroups as per the patient age, WHO grade and histological stage, and found that the risk score model could still pinpoint different outcome in subgroups. Particularly, the low-risk score group consistently represented better OS than the high-risk score group (Supplementary Fig. 2B-G). All these data clearly demonstrate the robustness of our EMTG risk score model.

To verify the accuracy of the EMTG model, we further validated it in external GEO cohort (GSE26193). Likewise, the OS patients (*n* = 107) were stratified into low- and high- risk score groups cut off by the median risk score. The K-M survival analysis revealed that the low-risk score group had better OS than the high-risk score group (Fig. 3G). The ROC analysis showed that the EMTG signature performed well in the prognostic prediction, with the AUC values of 0.734 (1-year), 0.697 (3-year), 0.669 (5-year), 0.715 (7-year), and 0.697 (9-year) respectively (Fig. 3H). Collectively, all these findings indicate that our EMTG risk score model could predict the survival status of OC patients with sufficient power.

### Assessment of the prognostic independency of the EMTG risk score model in OC

**Fig. 4:**
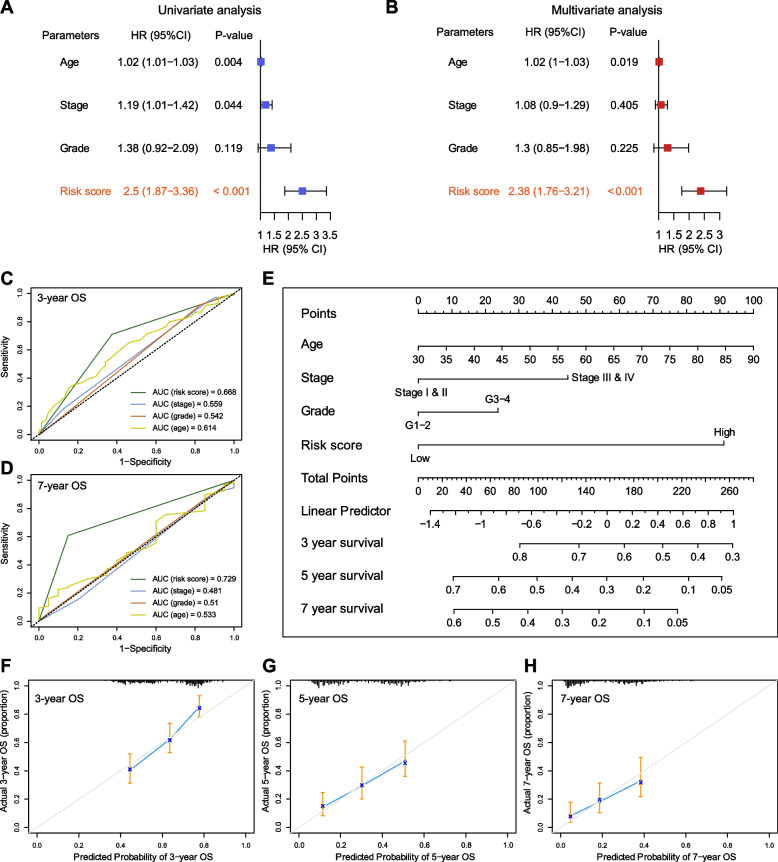
Assessment of the prognostic independency of EMTG risk score model in OC. **A**-**B** Univariate (**A**) and multivariate (**B**) Cox regression analysis showing that EMTG risk score was an independent factor for OS prediction in OC patients. **C-D** ROC curves showing the predictive efficiency of the EMTG risk score and clinical factors for 3- (**C**) and 5- (**D**) year survival rate in TCGA. **E** A nomogram showing the predicting probabilities of OC patients with 3-, 5-, and 7-year OS in TCGA. **F-H** Calibration plots showing the prediction of OC patients with 3- (**F**), 5- (**G**), and 7- (**H**) year OS in TCGA. X-axis indicating nomogram-predicted probability of survival; Y-axis indicating the actual survival

In view of the predictive ability of EMTG model in OC, we attempted to investigate whether the risk score model was an independent factor for the survival prediction of OC. Accordingly, we performed univariate and multivariate Cox regression analyses to evaluate the predictive power of OS-related parameters including age, stage, grade, and risk score. The univariate Cox analysis demonstrated that the age, stage, and risk score were statistically associated with the prognosis (*p* < 0.05) (Fig. 4A). The multivariate Cox analysis further indicated that only the age and risk score were consistently associated with the prognosis (*p* < 0.05) (Fig. 4B), and apparently, the risk score had the highest predictive power among all the parameters (Fig. 4C-D). Next, we tried to facilitate the clinical use of the EMTG model, and conducted a nomogram to predict the 3-, 5-, and 7- year survival status of OS patients (Fig. 4E). The calibration charts revealed the consistency of predictive and actual survival status for all the 3-, 5-, and 7- years (Fig. 4F-H). Thus, the EMTG signature is proved to be an independent factor for survival prediction, and its effectiveness is verified by multiple approaches.

### Investigation of molecular features in distinct EMTG risk score groups of OC

**Fig. 5:**
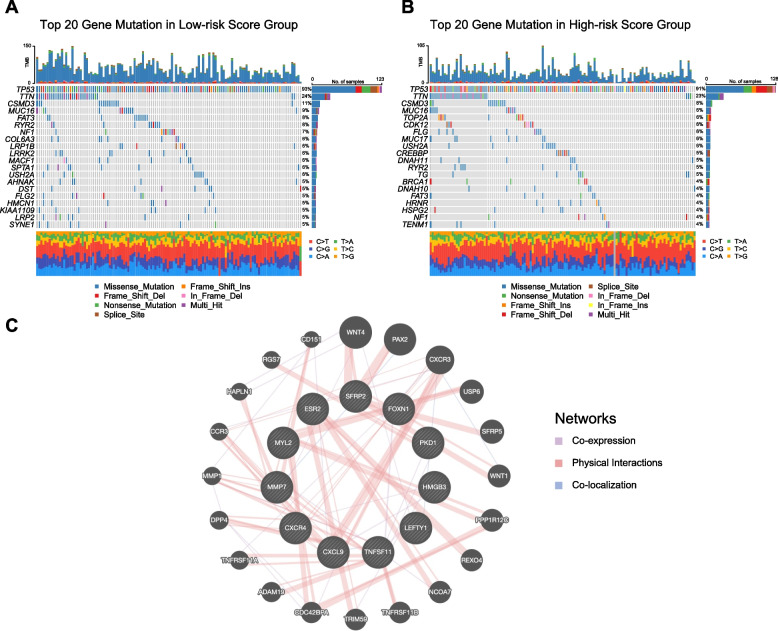
Investigation of molecular features in distinct EMTG risk score groups of OC. **A** The waterfall plots showing the top 20 gene mutations in the low-risk score group of OC. **B** The waterfall plots showing the top 20 gene mutations in the high-risk score group of OC. **C** A PPI diagram showing the protein-protein interaction of the 11-EMTG genes. The inner circle represents the 11 EMTG genes, and the outer circle represents the hub interactive genes calculated by GeneMANIA

To deeply explore the molecular difference between low- and high- risk score groups, we next studied genomic alterations by assessment of the DNA mutations. We found that genomic mutations existed in 96.97% of the low-risk score group and 94.33% of the high-risk score group. The top enriched genes were *TP53*, *TTN*, *CSMD3*, *MUC16*, which displayed no obvious difference between low- and high- risk score groups (Fig. 5A-B). However, it’s noted that the high-risk score group had more mutations on *TOP2A* (6%), *CDK12* (6%), and *BRCA1* (4%), by which may contribute to its poor prognosis.

To further understand how the 11 screened EMTGs contributed to OC development, we investigated their functions by protein-protein interaction (PPI) analysis. As shown, the 11 EMTGs were located in the center and surrounded by 20 hub proteins (Fig. 5C). Interestingly, we found that members of Wnt family (Wnt4, PAX2, Wnt1, SFRP5 etc.) were dramatically enriched in PPI network, which is widely known as a master regulator of EMT programs [36]. All these analyses suggest that genomic mutation and interactive network involving Wnt may contribute to the EMT regulation and OC progression.

### Identification of the immune landscape in distinct EMTG risk score groups of OC

**Fig. 6:**
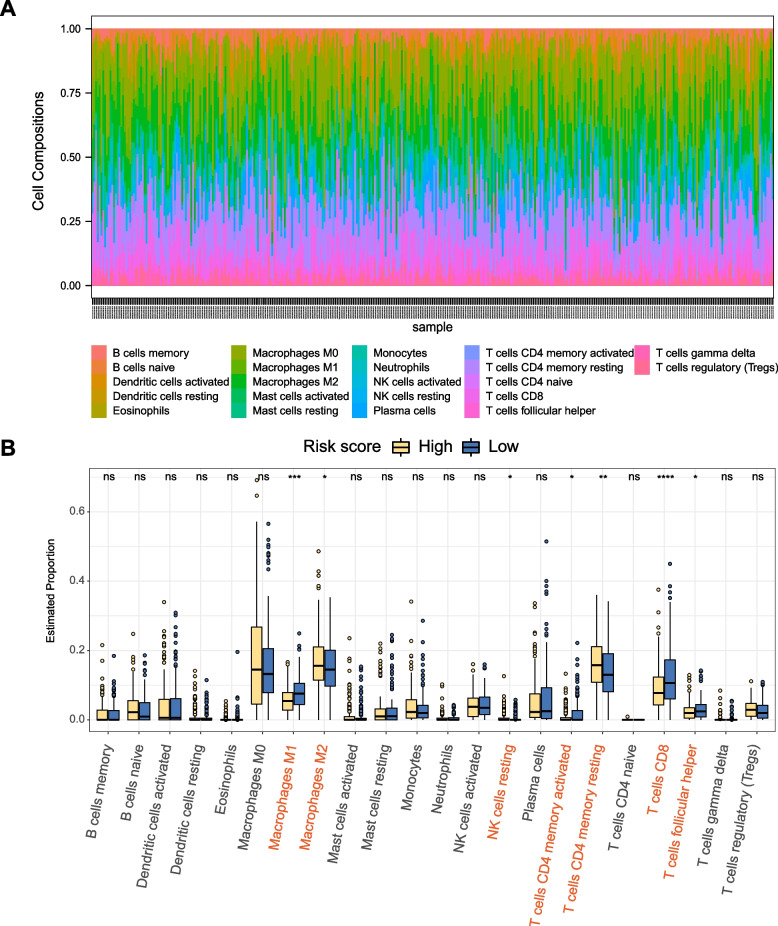
Identification of immune landscape in distinct EMTG risk score groups of OC. **A** A diagram showing the composition of 22 immune cells in OC cohort of TCGA. Colorful bars with different lengths represent individual immune cell proportions. **B** Box plots showing the difference of immune cell populations in low- and high-risk score groups in OC cohort of TCGA

The heterogenous immune microenvironment is an important orchestrator of OC development, and determines the prognosis [37]. Thus, we examined whether the different EMTG risk score groups displayed different immune microenvironment. Using the CIBERSORT algorithm, we evaluated alterations of 22 immune cells in TCGA-OC cohort. The distribution of each type immune cells was displayed in the diagram with variant abundant ratios for each sample (Fig. 6A). Apparently, there were 7 types of immune cells showed statistical difference between the low- and high- risk score groups (*p* < 0.05) (Fig. 6B). Compared with the low-risk score group, the high-risk score group held decreased levels of M1 macrophages, CD8 T cells, CD4 memory activated T cells and T follicular helper cells, together with increased M2 macrophages, resting NK cells, and CD4 memory resting T cells (Fig. 6B). These results suggests that the microenvironment of the high-risk score group is more immunosuppressive, which may contribute to its poor prognosis.

### Prediction of drug sensitivity in distinct EMTG risk score groups of OC

Chemotherapy is an indispensable OC management, however, chemoresistance results in treatment failure and tumor relapse [38]. To better understand the molecular mechanisms of chemoresistance in OC, we examined whether the EMTG model could predict drug sensitivity to chemotherapeutic agents. Thereby, we calculated the correlations of risk score to IC50 of drugs from the CellMiner database.

**Fig. 7:**
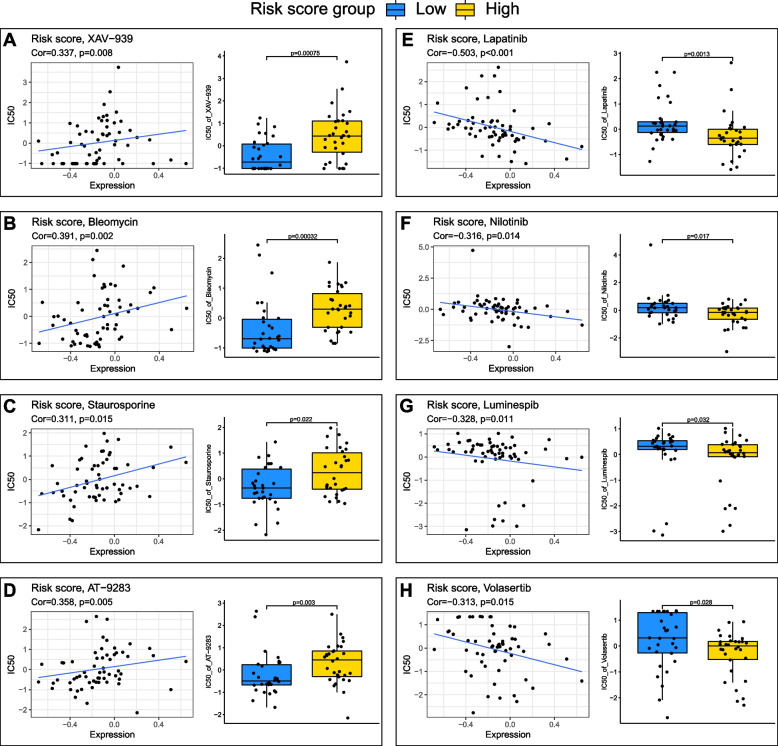
Prediction of drug sensitivity in distinct EMTG risk score groups of OC. **A**-**H** Scatter and box plots showing the correlations of EMTG risk score with IC50 of XAV-939 (**A**), Bleomycin (**B**), Staurosporine (**C**), AT-9283 (**D**), Lapatinib (**E**), Nilotinib (**F**), Luminespib (**G**), and Volasertib (**H**)

Results revealed different tendencies of the risk score to chemotherapy drugs. For instance, the IC50 of XAV-939, Bleomycin, Staurosporine and AT-9283 were increased as the elevated risk scores (Fig. 7A-B), while the IC50 of Lapatinib, Nilotinib, Luminespib and Volasertib were decreased as the elevated risk scores (Fig. 7E-H). Therefore, the former set of drugs were impactful for the low-risk score patients, and the later may be more effective for the high-risk score patients. Nevertheless, our EMTG risk score model is potential to play a role in the selection of chemotherapy drugs for individual OC patient.

## Discussion

OC is one of the most prevalent cancers for women in the world, characterized by its metastatic aggressiveness and unfavorable prognosis [13]. The rapid growth and invasion of OC is strongly regulated by epithelial-mesenchymal transition (EMT) [15]. In this study, we established a robust EMT-related gene model to predict the survival status of OC patients, which can be used in clinical management for prognosis surveillance.

EMT was originally identified by Betty Hay et al. in vertebrate embryonic development in 1960s [39, 40]. Over decades, current studies have established the underlying mechanisms by which EMT regulates OC malignant behaviors [41]. These experimental works provide the rationale for prognostic prediction of OC using EMT related genes. Actually, manipulating a series of genes to predict the prognosis is more accurate and definitive than only one single gene model [42]. Based on this assumption, previous studies have employed EMT genes to predict the prognosis of human cancers, including head and neck squamous cell cancer (HNSCC) [43], hepatocellular carcinoma [44], endometrial carcinoma [45], and colon adenocarcinoma [46]. But the predictive power of EMT genes in OC prognosis has not yet been evaluated. Therefore, we acquired the EMT gene expression profiles from TCGA and constructed an EMT gene-based risk score model to improve OC prognosis. Our risk score model was successfully validated by independent dataset.

During the model construction, we noticed that the set of EMT genes had low frequency of genomic mutations (Fig. 2). Instead, EMT gene expressions were dynamically regulated at the transcriptional level, supported by the fact that these genes were differentially expressed in the tumor tissues and normal controls. This point was further enhanced by previous findings showing that distinct EMT gene expression existed in morphologically indistinguishable samples from primary and primary and metastatic ovarian tumors [47]. The dynamic expressions of EMT genes are actually controlled by upstream regulations, including epigenetic modulation [48–50]. Therefore, understanding the molecular alteration of EMT genes will not only provide prognostic values, but also afford druggable targets in OC interventions.

In addition to provide a clinical tool for prognosis prediction, our EMT-related model also clarifies why the high-risk score group had rather poor survival. At the immune level, the immune infiltration analysis revealed that immunosuppressive cells were increased in the high-risk group (Fig. 6). The interconnective network of EMT signature genes with oncogenic signaling (e.g., Wnt) [36] exacerbates the malignancy in the high-risk group (Fig. 5). At the molecular level, the high-risk score group had elevated expressions of tumor enhancing factors, including *MMP7* [51], and *SFRP2* [52] (Fig. 3), which may synergistically promote tumor growth, proliferation, invasion and chemoresistance. For example, *MMP7* encodes matrix metalloproteinase-7 and facilitates the invasion and migration of ovarian tumor cells by remodeling the extracellular matrix [53, 54]. On the contrary, the low-risk score group had elevated expressions of tumor suppressing factors, including *ESR2* [55, 56]. For example, *ESR2* encode the estrogen receptor beta, and appeared to inhibit the EMT induced by E2 (17β-estradiol) in OC [55]. Moreover, elevated *ESR2* increases ERβ expression to block EMT and OC metastasis [56]. These experimental results are consistent with our bioinformatic analysis, showing that ESR2 expression is decreased as the risk score increasing. All these findings collaboratively address the reason for poor survival in high-risk score group of OC.

Nevertheless, our 11 EMT-gene based risk score model could improve the current prediction of survival status of OC patients. In fact, the predictive power of the clinical factors including age, WHO grade and histological stage are not satisfied, and our EMT-related risk score performed best compared with all these factors (Fig. 4). To the best of our knowledge, our study is the unique model using EMT genes in OC prognosis. Thus, our work will improve current understandings on gene set-based prognostic modeling, in parallel with facilitating the optimalization of chemotherapeutic strategies in clinical practice (Fig. 7).

## Conclusion

Collectively, our study established a novel EMT-based gene signature for OC, and revealed that the model was a reliable tool for prognostic prediction, with mechanistic indications of immune infiltration features and chemotherapeutic sensitivities.

## Supplementary Information

**Additional file 1: Figure S1.** The genomic mutation of EMT genes in OC cohort. The landscape of genomic mutations in OC cohort of TCGA, showing the variant classification (A), variant type (B), SNV class (C), variants per sample (D), variant classification (E), and top 10 mutated genes (F). **Figure S2.** Construction and exploration of the EMTG risk score model in OC; related to Fig. 3. (A) The procedure of LASSO Cox regression analysis for construction of the EMTG model in OC cohort of TCGA. (B-G) Kaplan–Meier analysis showing the distinct survival tendency in low- and high-risk score groups in sub-set of the TCGA cohort, including the young set (age ≤ 65, B), old set (age > 65, C), grade 1/2 set (D), grade 3/4 set (E), stage I/II set (F), and stage III/IV set (G).

## Data Availability

The datasets used and/or analyzed during the current study are available on the open repositories, including TCGA and GEO. The datasets used and/or analyzed during the current study are available from the corresponding author on reasonable request.

## Abbreviations

OC: Ovarian cancer
EMT: Epithelial-mesenchymal transition
EMTG: Epithelial-mesenchymal transition related gene
TCGA: The Cancer Genome Atlas
GO: Gene Ontology
KEGG: Kyoto Encyclopedia of Genes and Genomes
ROC: Receiver operating characteristic
AUC: Area under the ROC curve
PPI: Protein-protein interaction
