# Supplementary material for: An EMT-based gene signature enhances the clinical understanding and prognostic prediction of patients with ovarian cancers
Source: J Ovarian Res. 2023 Mar 13;16:51. doi: 10.1186/s13048-023-01132-2 (PMC10009944; doi:10.1186/s13048-023-01132-2)
Supplement: Supplementary file 1 — Additional file 1: Figure S1. The genomic mutation of EMT genes in OC cohort. The landscape of genomic mutations in OC cohort of TCGA, showing the variant classification (A), variant type (B), SNV class (C), variants per sample (D), variant classification (E), and top 10 mutated genes (F). Figure S2. Construction and exploration of the EMTG risk score model in OC; related to Fig. 3. (A) The procedure of LASSO Cox regression analysis for construction of the EMTG model in OC cohort of TCGA. (B-G) Kaplan–Meier analysis showing the distinct survival tendency in low- and high-risk score groups in sub-set of the TCGA cohort, including the young set (age ≤ 65, B), old set (age > 65, C), grade 1/2 set (D), grade 3/4 set (E), stage I/II set (F), and stage III/IV set (G). [file 13048_2023_1132_MOESM1_ESM.docx]

**Supplementary Materials for**

**An EMT-based Gene Signature Enhances the Clinical Understanding and Prognostic Prediction of Patients with Ovarian Cancers**

**Authors:** Qi-jia Li^1,#^, Zi-liang Wu^1,#^, Juan Wang^2^, Jing Jiang^1,*^, Bing Lin^1,*^

**Affiliations:**

^1^Hospital of Chengdu University of Traditional Chinese Medicine, Chengdu, 610072, China.

^2^Department of Public Health, School of Clinical Medicine, Chengdu University of Traditional Chinese Medicine, Chengdu, 610072, China.

^#^These authors contribute equally to this work.

***Correspondence:** Dr. Jing Jiang (13550060871@163.com) and Dr. Bing Lin (linbingcdutcm@163.com), No.39 Shi-er-qiao Road, Chengdu, 610072, Sichuan Province, China.

**This PDF includes,**

Supplementary Figures 1-2


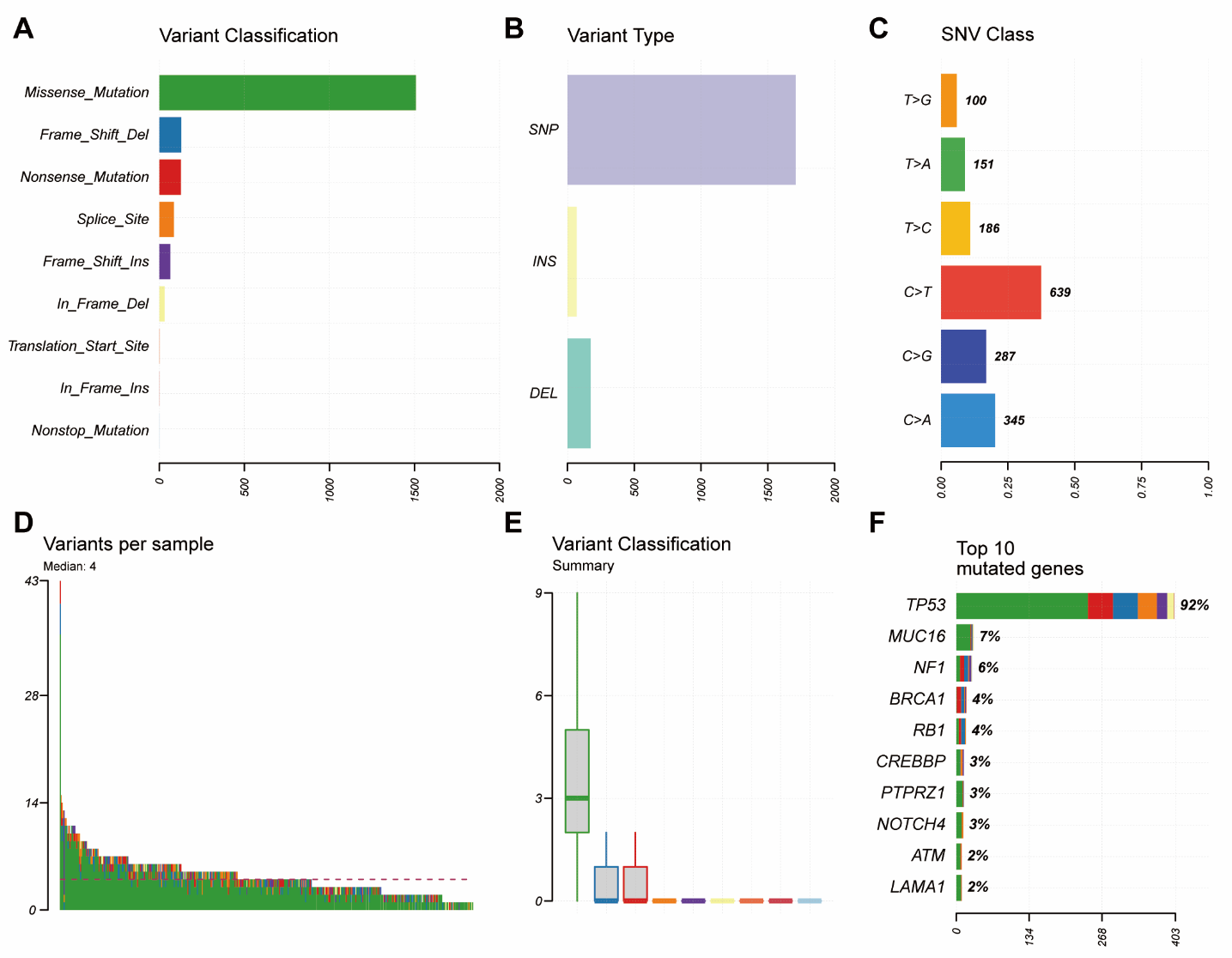
**Figure S1. The genomic mutation of EMT genes in OC cohort.**

The landscape of genomic mutations in OC cohort of TCGA, showing the variant classification **(A)**, variant type **(B)**, SNV class **(C)**, variants per sample **(D)**, variant classification **(E)**, and top 10 mutated genes **(F)**.


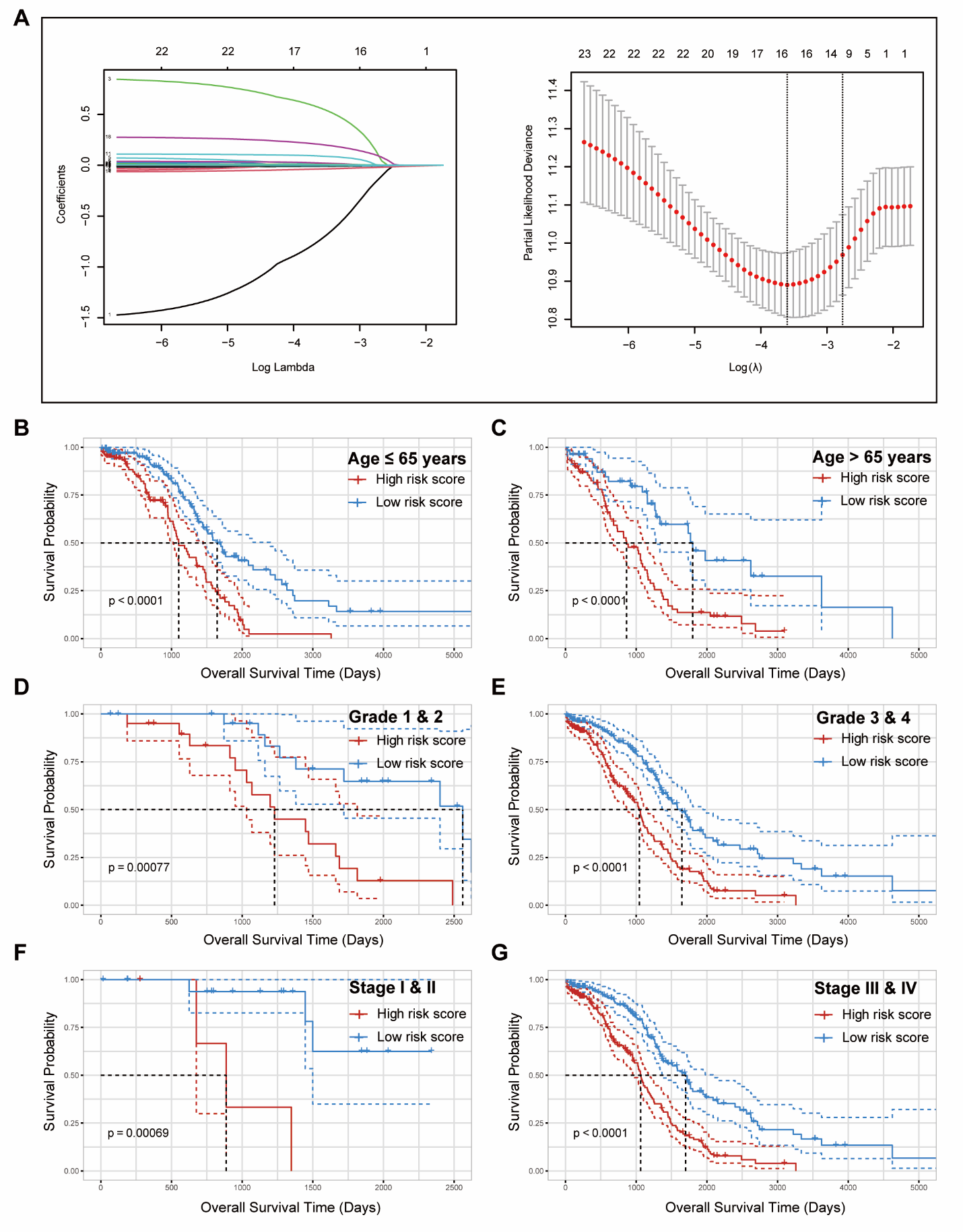
**Figure S2. Construction and exploration of the EMTG risk score model in OC; related to Figure 3.**

**(A)** The procedure of LASSO Cox regression analysis for construction of the EMTG model in OC cohort of TCGA. **(B-G)** Kaplan–Meier analysis showing the distinct survival tendency in low- and high-risk score groups in sub-set of the TCGA cohort, including the young set (age≤65, B), old set (age>65, C), grade 1/2 set (D), grade 3/4 set (E), stage I/II set (F), and stage III/IV set (G).
